# The multiple‐kinase inhibitor lenvatinib inhibits the proliferation of acute myeloid leukemia cells

**DOI:** 10.1002/ame2.12076

**Published:** 2019-09-03

**Authors:** Fan Feng, Xiaojuan Li, Ruisheng Li, Boan Li

**Affiliations:** ^1^ Center for Clinical Laboratory Fifth Medical Center General Hospital of Chinese PLA Beijing China; ^2^ Research Center for Clinical and Translational Medicine Fifth Medical Center General Hospital of Chinese PLA Beijing China; ^3^ Medical School of Chinese PLA Beijing China

**Keywords:** acute myeloid leukemia, lenvatinib, molecular targeting agents

## Abstract

**Background:**

Current chemotherapy for acute myeloid leukemia (AML) mainly involves cytotoxic agents such as doxorubicin (DNR), mitoxantrone (Mito) or 2‐aminopurine‐6‐thiol (6‐TG). However, because these agents are relatively ineffective, discovering other more effective drugs for AML treatment would be valuable.

**Methods:**

The in vitro antitumor effect of lenvatinib on AML cells was examined using the colorimetric MTT assay for assessing cell metabolic activity. AML cells mixed with Poloxamer 407 were injected into nude mice to form subcutaneous tumors. Tumor‐bearing mice received lenvatinib by oral administration. The antitumor effect of lenvatinib was established by measuring tumor volumes and weights.

**Results:**

Lenvatinib inhibited the growth of AML cells in a dose‐dependent manner. We used AML cells to establish subcutaneous tumor tissues by mixing the cell suspension with Poloxamer 407. Poloxamer 407 alone did not influence the subcutaneous growth of AML cells. Treatment of lenvatinib inhibited in vivo tumor growth of AML cells.

**Conclusion:**

The multiple‐kinase inhibitor lenvatinib inhibits the in vitro proliferation of AML cells, and restricts the in vivo growth of AML tumors.

## 1 INTRODUCTION

Acute myeloid leukemia (AML) is hematological malignancy that has been characterized by infiltration of the bone marrow, blood and other hematopoietic tissues.^1^, ^2^ Current chemotherapeutic options for AML include cytotoxic agents such as DNR, Mito, etoposide and 6‐TG.^3^, ^4^ However, treatment of AML patients with these agents has not shown the expected promising outcomes. Due to the complex multiple causes of AML, it is hard to achieve tumor regression by simply administrating a single functional cytotoxic agent.^5^

Over the last decade, molecule‐targeting agents have become widely recognized as “first choice” treatments for certain hardcore tumors, and researchers have now started to focus on treating one kind of tumor using kinase inhibitors approved to treat another.^6^, ^7^ A number of studies have provided insights into the genetic variability of AML and indicated potential targets for drug development. In addition to mutations found in genes including NPM1, CEBPA, DNMT3A, TET2, RUNX1, ASXL1, IDH2, and MLL, dysfunctional receptor tyrosine kinases (RTKs) such as Flt, VEGFR or c‐Kit have been shown to be drivers of the progress of AML.^5^, ^8^ Therefore, it would be valuable to discover whether effective existing kinase inhibitors restrict AML tumors.

Lenvatinib is a newly approved multi‐targeted kinase inhibitor.^9^ Oral administration of lenvatinib can repress the proliferation or metastasis of human malignancies by inhibiting the activation of RTKs.^10^, ^11^ In this work, we found lenvatinib inhibited the growth of AML cells in vitro. By mixing AML cells with Poloxamer 407, we were able to establish subcutaneous AML tumors in nude mice. Using this tumor model, we found that treatment of lenvatinib inhibited in vivo tumor growth of AML cells.

## 2 MATERIALS AND METHODS

### 2.1 Cell lines and agents

MHCC97‐H (a hepatocellular carcinoma cell line), and the AML cell lines Kg‐1 and Kg‐1a were purchased from the Type Culture Collection of the Chinese Academy of Sciences, Shanghai, People's Republic of China, a culture collection center of the Chinese government. OCL‐AML‐5 (cat. no. BNCC339352), ME‐1 (cat. no. BNCC340048) and OCI‐AML3 (cat. no. BNCC341618) were purchased from the BeNa Culture Collection Corporation. The antitumor agent lenvatinib was purchased from Selleck Corporation. Poloxamer 407 was a gift from Dr Tao Wang at the Institute of Pharmacology and Toxicology, Academy of Military Medical Sciences.^12^

### 2.2 Cell culture and proliferation inhibition testing

AML cells were cultured in Dulbecco's Modified Eagle's medium (DMEM, Thermo Fisher Corporation) with 10% fetal bovine serum (FBS, Thermo Fisher Corporation) at 37°C under 5% CO_2_. The use of the cell lines was approved by the Ethics Committee, The General Hospital of Central Theater Command, Chinese People's Liberation Army. The antitumor agent lenvatinib was first dissolved in the organic solvent dimethyl sulfoxide (DMSO), and this solution was then diluted with DMEM. To examine cell proliferation inhibition, 2000 cells were seeded into 96‐well culture plates. The cells were then treated with different concentrations of lenvatinib (1, 0.3, 0.1, 0.03, 0.01, 0.003 or 0.00 μmol/L) for 48 hours. Next cell metabolic activity was measured by MTT assay. Briefly, MTT assays were performed using the following methods. MTT reagent (50 mg/mL) was added to the wells of a 96‐well culture plate and after about 4 hours the plates were centrifuged to attach the cells to the bottom of the plates. The supernatant was discarded and about 200 μL DMSO was added to each well. After standing for about 10 minutes, the plates were shaken to break up the cells. The plates were then centrifuged to remove the resulting foam and screened using a plate‐reader to obtain the absorbance of samples at a wavelength of 490 nm. The rate of inhibition by lenvatinib of AML cell proliferation was calculated as {[(48 hour time point OD 490 nm of control group)−(0 hour time point OD 490 nm of control group)] −[(48 hour time point OD 490 nm of lenvatinib group)−(0 hour time point OD 490 nm of lenvatinib group)]}/[(48 hour time point OD 490 nm of control group)−(0 hour time point OD 490 nm of control group)] × 100%.

### 2.3 Subcutaneous growth of AML cells

The animal experiments were approved by the Institutional Animal Care and Use Committee of The Fifth Medical Center (former name: The 302nd Hospital of Chinese PLA), General Hospital of Chinese People's Liberation Army (the ID of certification: IACUC‐2019‐001, Figure S1). All the animal studies were conducted in accordance with the UK Animals (Scientific Procedures) Act of 1986 and the associated guidelines. AML cells or MHCC97‐H cells were cultured and resuspended in physiological saline. Next, cell suspensions or cell suspensions containing 2% Poloxamer 407 were subcutaneously injected into nude mice. The concentration of cells in the cell suspension was 10^7^ cells/mL. About 0.2 mL cell suspension per site was injected subcutaneously into nude mice at various sites. Three to four days’ after injection, the mice received 1, 0.5, 0.2 or 0.1 mg/kg lenvatinib by oral administration. The mice received lenvatinib treatment once per 2 days. After 10 treatments (about 20‐21 days), the mice were harvested and the volumes or weights of subcutaneous tumor tissues were examined. The tumor volumes of subcutaneous tumor tissues were calculated as tumor length × tumor width × tumor width/2.^13^, ^14^ The inhibition rates of lenvatinib on the subcutaneous growth of AML cells were calculated as [(control group tumor volumes)−(lenvatinib group tumor volumes)]/(control group tumor volumes) × 100%; [(control group tumor weights)−(lenvatinib group tumor weights)]/(control group tumor weights) × 100%.

### 2.4 Subcutaneous tumor tissue shape regularity

Photographs of subcutaneous tumors were quantitatively analyzed to determine the length of the long axis of the tumor, and the area of the perfect circle (the total number of pixels) having the diameter of the long axis was calculated. At the same time, the tumor tissue was delineated by marking the edge of the tumor on the photograph and the total area of the tumor tissue (total number of pixels) was calculated. The ratio of the total area of the long‐axis circle to the total area of the delineated tumor reflected the regularity of the shape of the tumor tissue: the closer the ratio to 1, the more regular the shape of the tumor tissue.

### 2.5 Statistical analysis

Statistical analysis was performed using a Bonferroni correction with two‐way analysis of variance and *P* < .05 were considered as statistically significant between groups. The statistical analysis was performed using SPSS software (IBM Corporation) and the IC_50_ values of lenvatinib on AML cells were calculated using Origin software (OriginLab Corporation). The concentrations and the inhibition rates were analyzed with Origin software using the Sigmoidal Fit methods.

## 3 RESULTS

### 3.1 Lenvatinib inhibits the proliferation of cultured AML cells in a dose‐dependent manner

**Figure 1:**
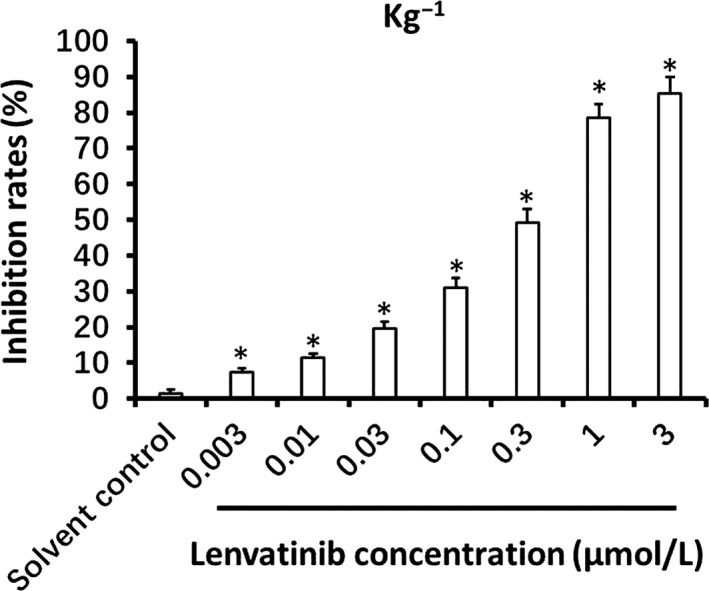
The anti‐tumor effect of lenvatinib on Kg‐1 cells. Kg‐1 cells treated with the indicated concentrations of lenvatinib were analyzed by MTT assay. Inhibition rates of lenvatinib on Kg‐1 cells are shown as means ± SD. *P < .05, lenvatinib treatment group versus solvent control group

In order to study the effect of lenvatinib on AML cells, we first examined whether it affected the proliferation of the target cells. We treated in vitro cultured AML Kg‐1 cells with incremental concentrations of lenvatinib for 48 hours, followed by MTT assay. We then observed whether lenvatinib inhibited the proliferation of other AML cells in a dose‐dependent manner as expected (Figure 1; Table 1). Our data show that lenvatinib inhibits the proliferation of cultured AML cells in a dose‐dependent manner.

**Table 1:** The IC_50_ values (μmol/L) of lenvatinib on cultured AML cell lines

Cell line	IC_50_ values (μmol/L) of lenvatinib
Kg‐1	0.26 ± 0.03
Kg‐1a	0.30 ± 0.05
OCL‐AML‐5	0.22 ± 0.01
ME‐1	0.35 ± 0.06
OCI‐AML3	0.25 ± 0.05

Abbreviation: AML, acute myeloid leukemia.

### 3.2 Establishing an AML subcutaneous tumor model in nude mice

**Figure 2:**
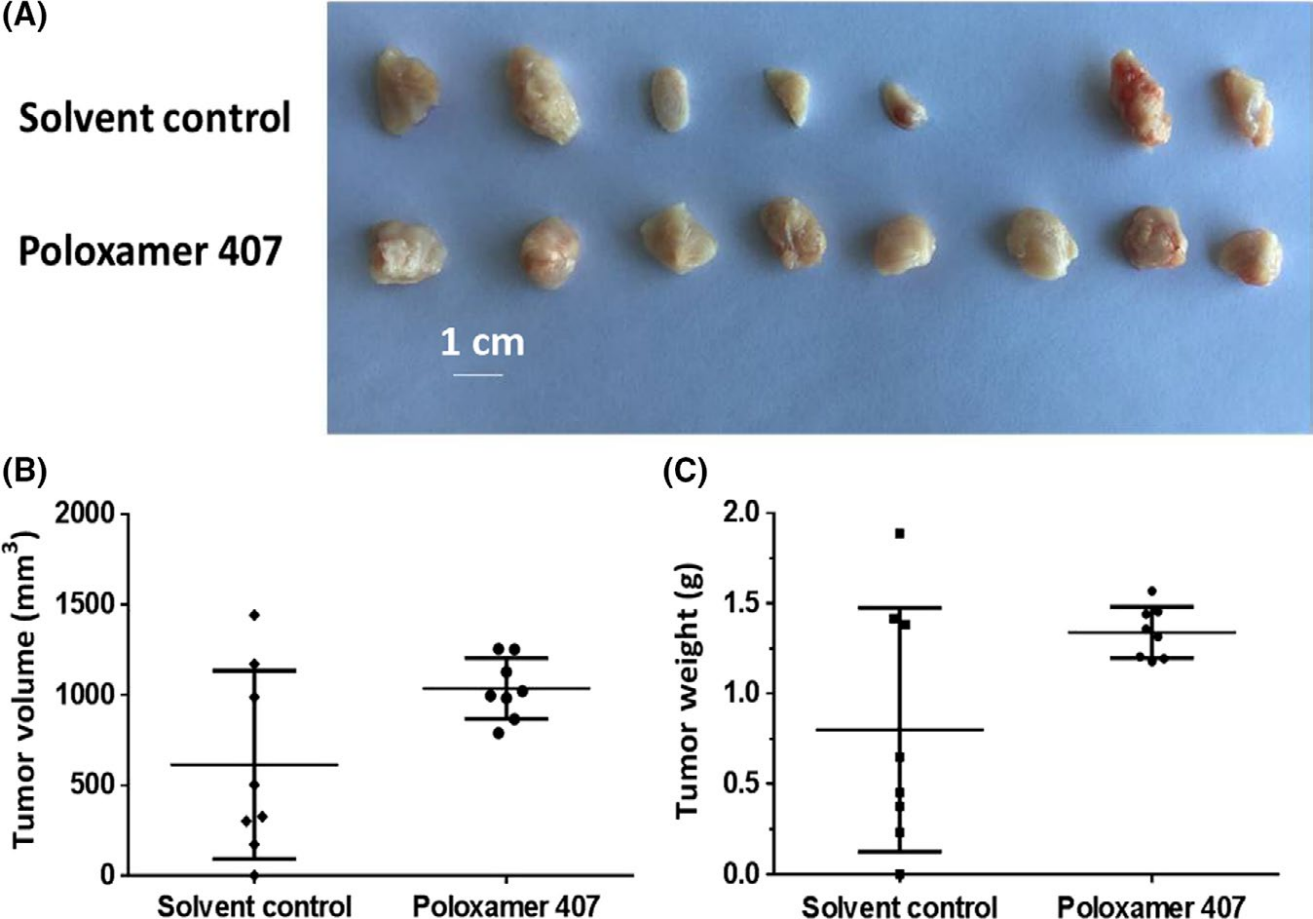
Kg‐1 cells form subcutaneous tumors in nude mice. Kg‐1 cells were cultured and then prepared as cell suspensions in physiological saline or physiological saline with 2% Poloxamer 407. Cell suspensions were injected into nude mice to form subcutaneous tumors. Results are shown as images of tumor tissues (A), tumor volumes (B), or tumor weights (C)

To overcome the difficulty that AML cells rarely form regular solid tumors subcutaneously and to enable measurement of tumor growth, we attempted to establish a subcutaneous growth model for these hematological malignant cells. As shown in Figure 2, injection of a cell suspensions of Kg‐1 cells into subcutaneous sites in nude mice resulted in the formation of uneven and irregular shaped tumors (Figure 2A). We next added 2% Poloxamer 407 to the Kg‐1 cell suspension before subcutaneously injecting the nude mice. The size of the subcutaneous tumors became even and the shapes were homogenous (Figure 2; Table 2). Our data show that this AML subcutaneous tumor model can be used for further evaluation of lenvatinib.

**Table 2:** Shape homogeneity of subcutaneous tumors formed by AML cell lines

Tumor no.	Kg‐1		Kg‐1a		OCL‐AML‐5		ME‐1		OCI‐AML3	
	Control	Polo	Control	Polo	Control	Polo	Control	Polo	Control	Polo
1	1.62	1.33	2.45	1.34	1.24	1.27	1.75	1.2	1.35	1.12
2	1.89	1.15	1.13	1.11	1.45	1.35	1.56	1.11	1.12	1.22
3	1.52	1.28	1.88	1.22	2.01	1.11	1.35	1.19	0	1.31
4	2.34	1.38	1.76	1.31	1.86	1.48	1.48	1.35	1.73	1.13
5	1.52	1.21	0	1.18	1.25	1.56	2.22	1.51	2.34	1.15
6	0	1.24	1.66	1.25	1.44	1.21	1.91	1.17	1.54	1.21
7	2.17	1.17	1.54	1.22	1.95	1.33	2.21	1.13	0	1.29
8	2.16	1.24	2.22	1.19	2.05	1.44	1.65	1.28	2.14	1.32

Abbreviations: Control, solvent control; Polo, Poloxamer 407.

### 3.3 Lenvatinib inhibits the subcutaneous growth of AML cells

**Figure 3:**
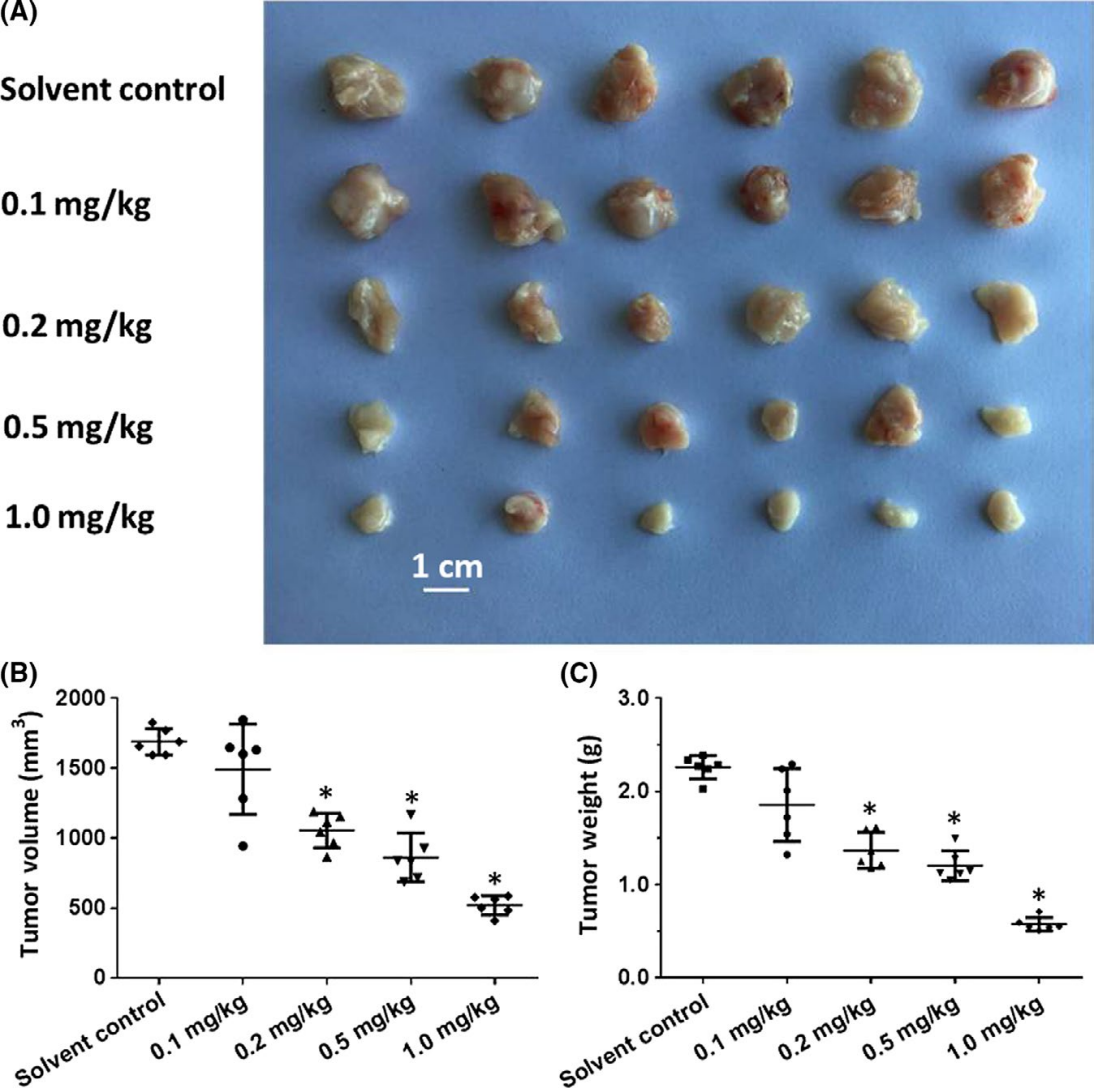
The anti‐tumor effect of lenvatinib on subcutaneous tumors formed by Kg‐1 cells. Kg‐1 cells were cultured and then prepared as cell suspensions in physiological saline with 2% Poloxamer 407. Cell suspensions were injected into nude mice to form subcutaneous tumors. Four to 5 d after injection, the mice received lenvatinib by oral administration once per 2 d. After 10 treatments, the mice were harvested. The results are shown as images of tumor tissues (A), tumor volumes (B), or tumor weights (C). *P < .05 versus lenvatinib group with solvent control group

The in vivo antitumor effect of lenvatinib on AML cells was then examined. In our newly established tumor models, we found that oral administration of lenvatinib inhibits the subcutaneous growth of Kg‐1 cells in a dose‐dependent manner. Moreover, treatment with lenvatinib also inhibits the subcutaneous growth of other AML cell types (Figure 3; Table 3). Thus, our results suggest lenvatinib can inhibit in vivo AML tumors.

**Table 3:** The IC_50_ values (μmol/L) of lenvatinib on subcutaneous growth of AML cell lines

Cell line	IC_50_ values (mg/kg) of lenvatinib
Kg‐1	0.33 ± 0.10
Kg‐1a	0.45 ± 0.08
OCL‐AML‐5	0.25 ± 0.05
ME‐1	0.30 ± 0.07
OCI‐AML3	0.35 ± 0.09

Abbreviation: AML, acute myeloid leukemia.

### 3.4 Poloxamer 407 alone does not influence the survival or growth of AML cells

**Figure 4:**
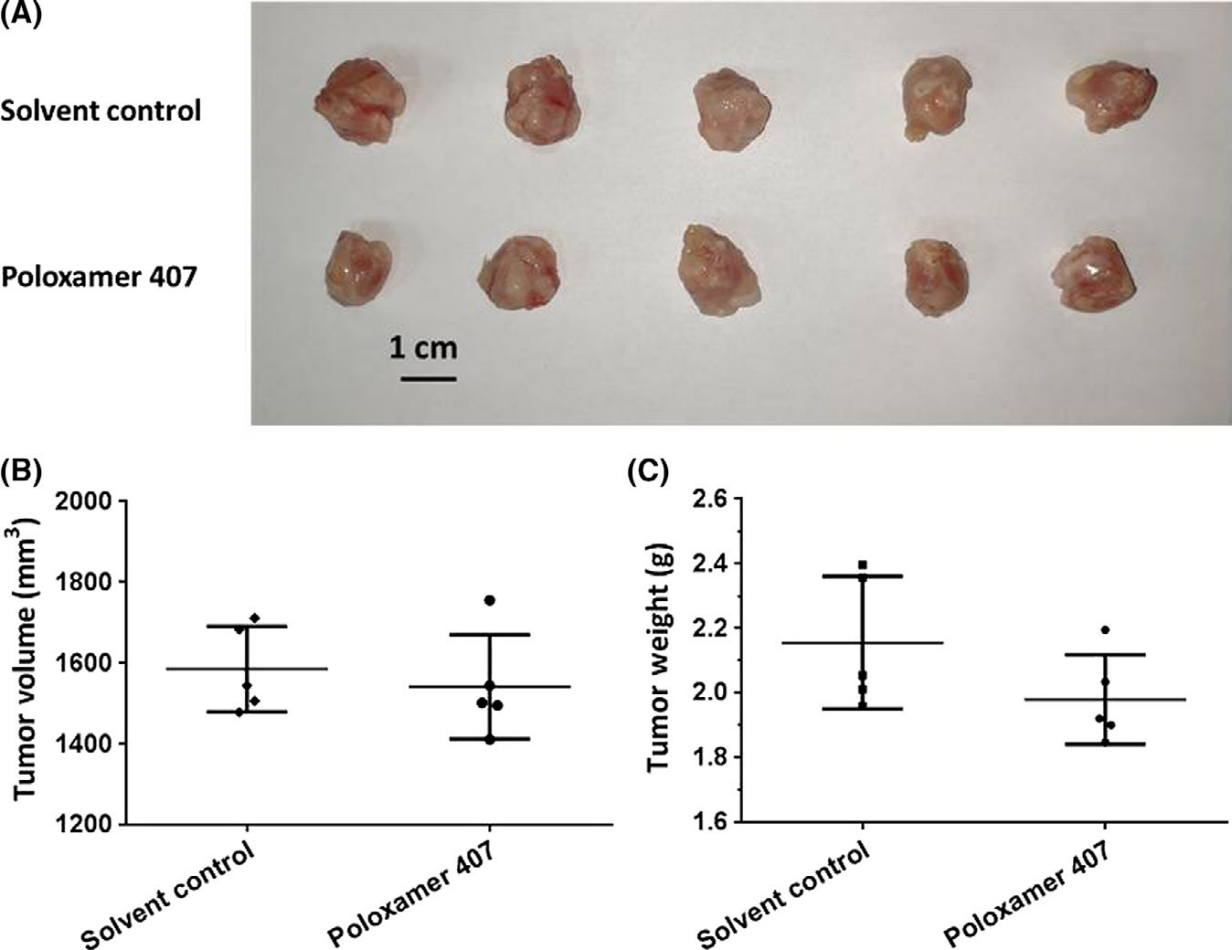
Poloxamer 407 alone has no antitumor effect on subcutaneous growth of MHCC97‐H cells in nude mice. MHCC97‐H cells were cultured and then prepared as cell suspensions in physiological saline or physiological saline with 2% Poloxamer 407. Cell suspensions were injected into nude mice to form subcutaneous tumors. The results were shown as images of tumor tissues (A), tumor volumes (B), or tumor weights (C)

**Figure 5:**
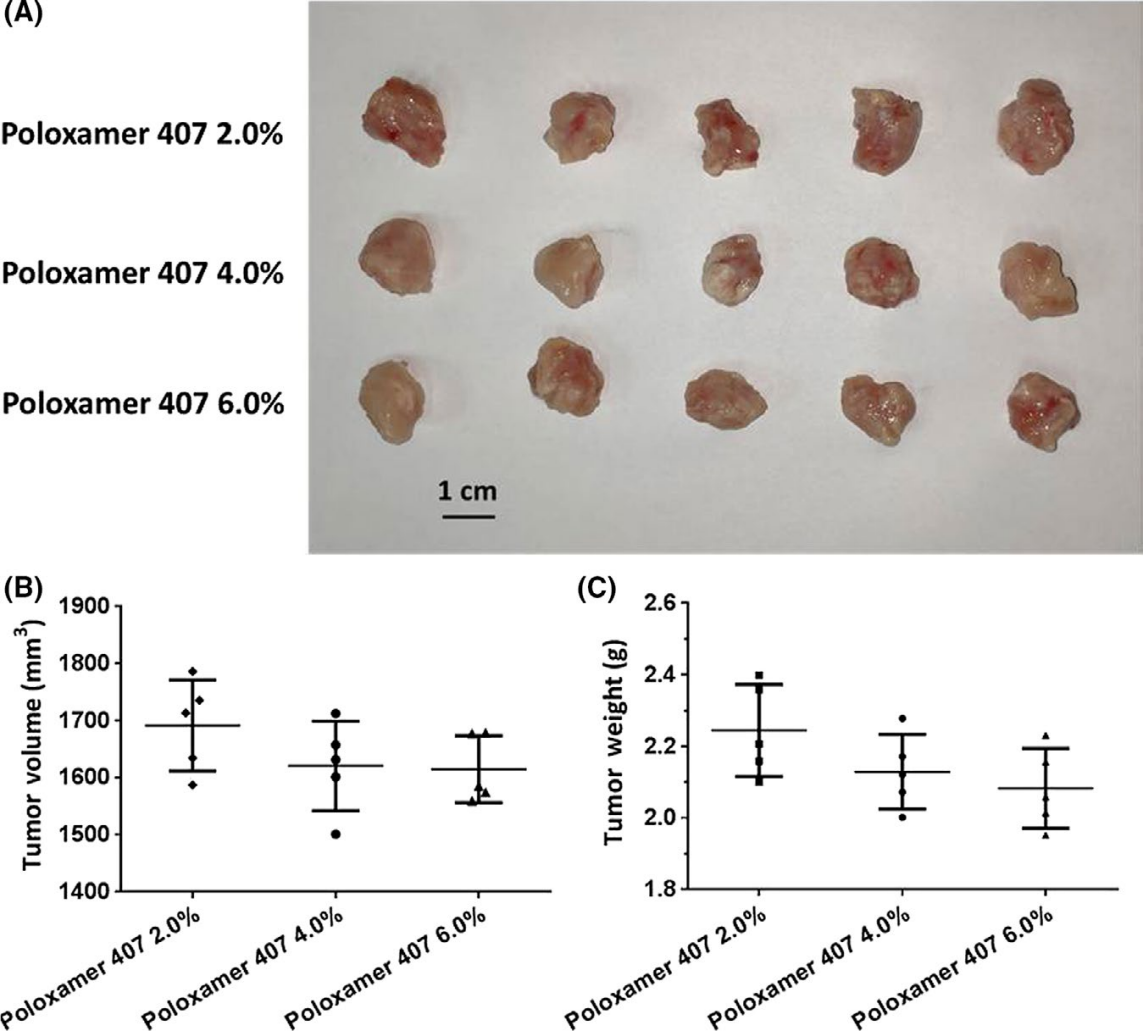
Poloxamer 407 alone as no anti‐tumor effect on subcutaneous growth of Kg‐1 cells in nude mice. Kg‐1 cells were cultured and then prepared as cell suspensions in physiological saline with 2%, 4%, or 6% Poloxamer 407. Cell suspensions were injected into nude mice to form subcutaneous tumors. The results were shown as images of tumor tissues (A), tumor volumes (B), or tumor weights (C)

To examine the effect of Poloxamer 407 on the subcutaneous growth of AML cells, we firstly established subcutaneous tumors using MHCC97‐H cells, a highly aggressive HCC cell line, with/without 2% Poloxamer 407 (Figure 4). We found that this concentration of Poloxamer 407 did not affect the subcutaneous growth of HCC cells (Figure 4). Next, cell suspensions of AML cells with increasing concentrations of Poloxamer 407 were injected into nude mice. As shown in Figure 5, there was no significant difference between the subcutaneous tumor tissues from the nude mice injected with cell suspensions of AML cells containing the different concentrations of Poloxamer 407. Therefore, Poloxamer 407 alone does not influence the survival or growth of AML cells.

## 4 DISCUSSION

The overall 5‐year survival of AML patients still remains very low (about 26%), especially in aged patients (5%‐15%).^1^, ^2^ Routine AML therapy relies on patient selection for induction chemotherapy, timing of stem cell transplantation and optimal supportive care.^1^, ^2^ Recently, the chemotherapy agents doxorubicin (DNR), mitoxantrone (Mito), etoposide or 2‐aminopurine‐6‐thiol (6‐TG) have become the preferred choices for AML treatment.^15^, ^16^, ^17^ Meanwhile, molecular‐targeted agents, eg midostaurin or enasidenib, have been approved for AML treatment and brought new hope for AML patients.^18^, ^19^, ^20^, ^21^ Midostaurin, developed by Novartis Corporation, targets FLT3, c‐KIT, PDGFRB, VEGFR‐2 or protein kinase C; enasidenib, developed by Agios Corporation and Celgene Corporation, targets isocitrate dehydrogenase 2 (IDH2) to specifically inhibit the function of IDH2 mutants, reduces 2‐HG levels and reverses genomic hypermethylation status of AML.^18^, ^19^, ^20^, ^21^ However, as clinical practical experience is accumulating, these agents have not led to the hoped‐for improvements in the survival of or prognosis for AML patients, suggesting that kinase inhibition may exert mild effects at the beginning of tumor development but lose its effect early on. Thus, understanding the reason(s) for this outcome and developing novel therapeutic methods is critically important.

Genetic studies have revealed a large number of aberrations in AML patients.^1^, ^2^ Prognostic gene‐gene interactions have also showed mutations in NRAS, DNMT3A, NPM1, DNMT3A, and IDH2R140.^1^, ^2^ The mutated genes/proteins lead to the dysfunction of multiple kinases in AML, which may explain the failure of single kinase inhibitor administration: the dysfunctional kinase pathways compensate each other when pharmaceutical inhibition occurs. However, use of multiple inhibitor combinations is not easily accomplished due to their superposed toxicity, which may overwhelm their antitumor effects. Thus, we focused on lenvatinib, which has a high affinity for RTKs, eg VEGFR2/KDR, VEGFR3/FLT4, VEGFR1/FLT1, PDGFRβ, FGFR1, PDGFRα or c‐Kit.^22^ Given that several target kinases such as PDGFRα and c‐Kit have been shown to play important roles in AML cell proliferation, we anticipated that lenvatinib should have a considerable anti‐AML effect. In the present work, our results showed that lenvatinib inhibited the in vitro and in vivo growth of AML cells in a dose‐dependent manner. Our research expands the understanding of molecular targeting therapy for AML.

In addition, there are few methods available for anti‐tumor research related to blood tumors. Isolation of AML primary cell lines or in vitro culture using AML cell lines can be used to detect the antitumor activity of drugs. However, in vivo experiments are not easily performed. Unlike cell lines derived from solid tumor tissues such as HCC cells, AML cells derive from blood tumor cells and do not easily form tumor tissue under the skin of nude mice. Traditional animal experiments often involve inoculating AML cells into the circulation system of immunodeficient animals (eg nude mice), extracting blood samples from some of the animals, and then counting AML cells in the blood samples. The total number of AML cells in the whole animal (tumor cell load) is calculated from the number of blood samples taken and the total blood volume or the body weights. This method is not precise enough and has a large error.

In the present work, adding a small amount (2%) of a polymer material, Poloxamer 407, to the cell suspension assisted AML cells to form subcutaneous tumor tissues in nude mice. Quantitative analysis of the anti‐tumor activity of drugs could then be achieved by weighing and measuring volumes of subcutaneous tumor tissues. There are many kinds of tumors of the blood system, and cells may form solid tumors in organs, eg lymph nodes. This makes the method used in the present work more relevant to blood tumor research. Poloxamer 407 is a biodegradable pharmaceutical excipient that is safe to use on its own.^12^ Results showed that Poloxamer 407 alone did not affect the subcutaneous growth of cells in nude mice.

The growth of tumor cells in tumor tissues is affected by the microenvironment. Therefore, it would be valuable to examine whether the formation of solid tumor tissue changes the characteristics of AML cells. Leukemia cells such as AML are mainly present in the human body in circulating system tumors. AML cells cultured in vitro also exhibit suspension growth. In this study, possible mechanisms for the action of Poloxamer 407 include: (a) playing a role in immobilizing AML cells in the subcutaneous position and (b) mimicking the interaction of the extracellular matrix with AML cells, but without the effects of the growth factors found in the extracellular matrix, and thus not affecting the biological features of the cells.

## CONFLICT OF INTEREST

None.

## AUTHOR CONTRIBUTIONS

All authors made substantial contributions to the design and conception of the study and acquisition, analysis or interpretation of data. All authors took part in either drafting or revising the manuscript. All authors also read and approved the final version manuscript for publication, and agree to be accountable for all aspects of the work by ensuring that questions related to the accuracy or integrity of any part of the work are appropriately investigated and resolved.
