# Supplementary figures and images for: The multiple‐kinase inhibitor lenvatinib inhibits the proliferation of acute myeloid leukemia cells
Source: Animal Model Exp Med. 2019 Sep 3;2(3):178–84. doi: 10.1002/ame2.12076 (PMC6762047; doi:10.1002/ame2.12076)

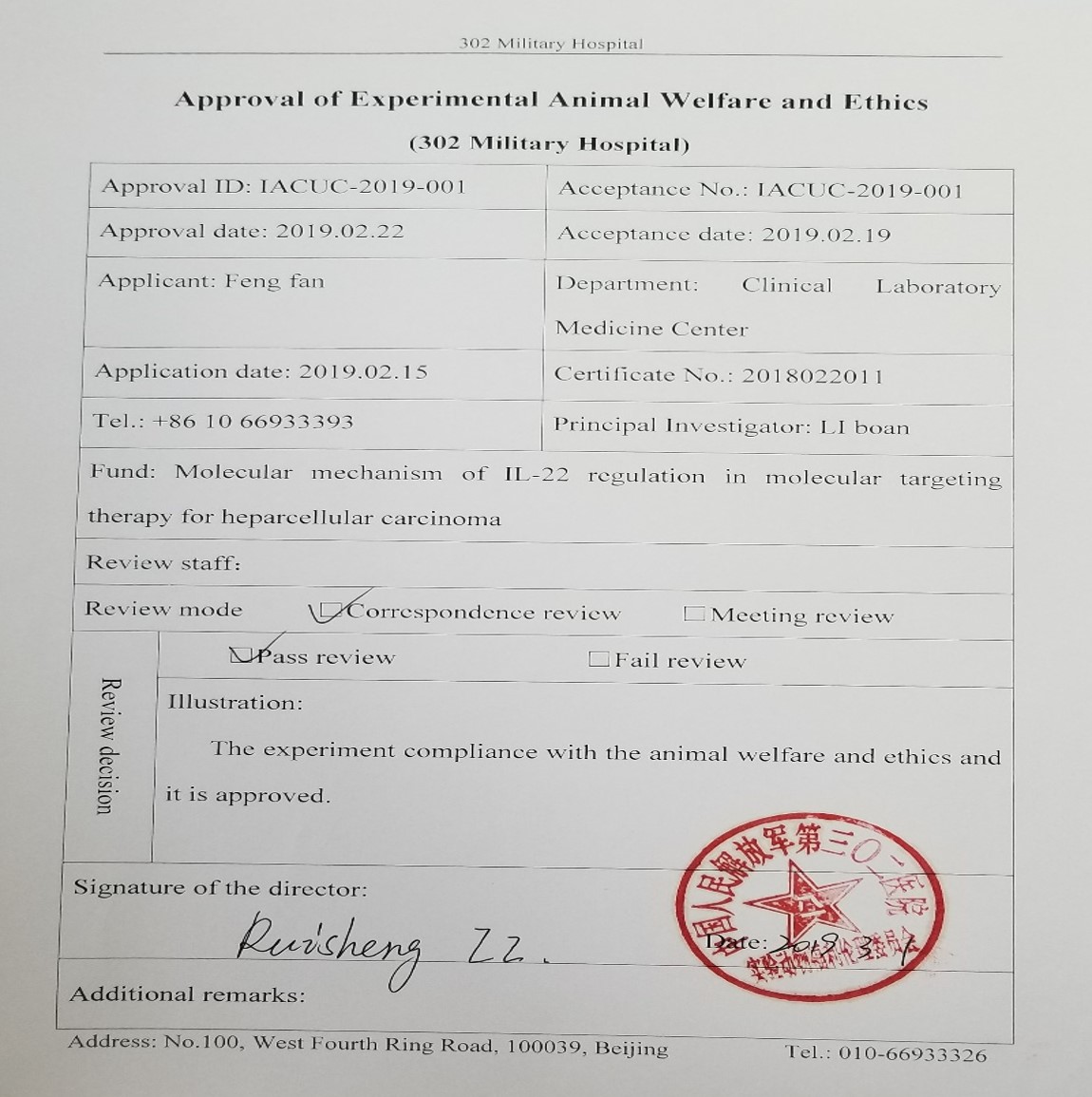

Supplement: Supplementary file 1 [file AME2-2-178-s001.jpg]
